# The Reservoir of Persistent Human Papillomavirus Infection; Strategies for Elimination Using Anti-Viral Therapies

**DOI:** 10.3390/v14020214

**Published:** 2022-01-22

**Authors:** Ke Zheng, Nagayasu Egawa, Aslam Shiraz, Mayako Katakuse, Maki Okamura, Heather M. Griffin, John Doorbar

**Affiliations:** 1 Department of Pathology, University of Cambridge, Cambridge CB2 1QP, UK; kz286@cam.ac.uk (K.Z.); ne259@cam.ac.uk (N.E.); mas202@cam.ac.uk (A.S.); hmg36@cam.ac.uk (H.M.G.); 2 Kyoto R&D Centre, Maruho Co., Ltd., Kyoto 600-8813, Japan; katakuse_dzn@mii.maruho.co.jp (M.K.); okamura_emq@mii.maruho.co.jp (M.O.)

**Keywords:** persistent infection, HPV, anti-viral therapy, basal epithelial homeostasis

## Abstract

Human Papillomaviruses have co-evolved with their human host, with each of the over 200 known HPV types infecting distinct epithelial niches to cause diverse disease pathologies. Despite the success of prophylactic vaccines in preventing high-risk HPV infection, the development of HPV anti-viral therapies has been hampered by the lack of enzymatic viral functions, and by difficulties in translating the results of in vitro experiments into clinically useful treatment regimes. In this review, we discuss recent advances in anti-HPV drug development, and highlight the importance of understanding persistent HPV infections for future anti-viral design. In the infected epithelial basal layer, HPV genomes are maintained at a very low copy number, with only limited viral gene expression; factors which allow them to hide from the host immune system. However, HPV gene expression confers an elevated proliferative potential, a delayed commitment to differentiation, and preferential persistence of the infected cell in the epithelial basal layer, when compared to their uninfected neighbours. To a large extent, this is driven by the viral E6 protein, which functions in the HPV life cycle as a modulator of epithelial homeostasis. By targeting HPV gene products involved in the maintenance of the viral reservoir, there appears to be new opportunities for the control or elimination of chronic HPV infections.

## 1. Introduction—‘Outlining the Need for HPV Antiviral Therapies’

Human papillomaviruses (HPV) comprise more than 200 types that are grouped into five genera (α, β, γ, µ and η) with tropisms for skin and mucosal epithelial sites. Infection leads to characteristic HPV type-specific disease pathologies, ranging from benign hyperproliferative lesions to inapparent or asymptomatic infections that can, in certain individuals, progress to high-grade neoplasia and invasive cancer. As a group, these viruses have evolved to infect and thrive at many different body sites by adapting to local epithelial conditions, which vary not only between cutaneous and mucosal epithelia, but also amongst epithelial appendages, such as hair follicles and sweat glands, with each of these sites providing distinctive epithelial niches and mechanisms of site-specific immune regulation [1,2].

Studies on the “high-risk” HPV (hrHPV) types, which are implicated in the development of cervical cancer, have, in the last few decades, led to the development of HPV nucleic acid tests useful in cervical screening, as well as to the development of prophylactic vaccines [3]. The widespread implementation of such vaccines has clear potential [4], especially when used alongside effective cervical screening programmes, and there is now considerable effort devoted to the use of both approaches in many countries. There remains, however, no effective strategy for the treatment of low- or high-grade cervical lesions other than surgery, with the former being managed in the community by repeat screening, until immune control or disease clearance is achieved spontaneously, or progression to high-grade neoplasia necessitates surgical intervention. These follow-up examinations pose a significant psychological burden on the patient. For women who require treatment, the depth of surgical excision influences the risk of cervical incompetence and pre-term labour—an issue which is compounded by the fact that most women requiring excisional treatment are of child-bearing age. Less traumatic treatment regimens, such as cryotherapy [5] have been trialled, but unfortunately do not ensure a depth of treatment necessary for lesion ablation. The low-risk HPV (lrHPV) types are typically associated with benign hyperproliferative lesions [6], and although not usually life-threatening, these can also be associated with significant morbidity. The incidence of new cases of genital warts per year in the UK is 0.16% [7], and the total cost to the healthcare system is £58.44 million/year [8]. Cutaneous HPV-associated lesions (such as common warts) typically affect young children who lack previous HPV exposure [9,10]. Irrespective of the epithelial target site, however, all lrHPV infections can be problematic in susceptible groups, such as Epidermodysplasia verruciformis (EV) patients, individuals who are immunosuppressed (e.g., HIV-infected and transplant patients), and those suffering from recurrent respiratory papillomatosis (RRP), where lrHPV-associated cancers may occur [11]. HPV-associated papillomas can be highly refractory to treatment, persisting for years, and pose a problem cosmetically, psychologically, and may affect day-to-day life depending on the extent of lesion spread and infection sites [12]. Whether caused by hrHPV or lrHPV, the fact that these infections are common and persistent suggests that a cost-effective treatment modality would attract significant attention from the clinical community.

## 2. The Development of Anti-HPV Pharmaceuticals—Basic Considerations and Approaches

**Figure 1:**
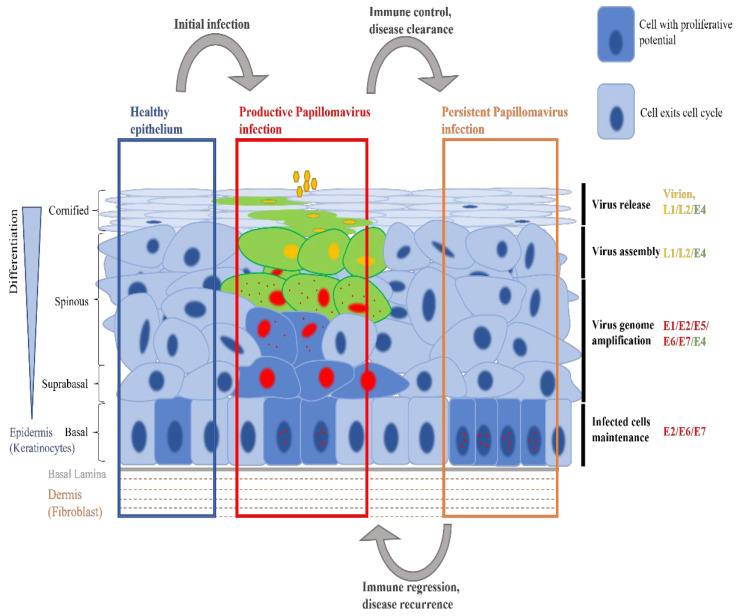
Human Papillomavirus Infection of Stratified Epithelium. In healthy epithelial tissue, a proportion of basal keratinocytes retain the potential to proliferate in order to maintain the multilayered epithelial structure. Following HPV infection, viral gene expression can alter the normal differentiation program of the cell to facilitate completion of the productive virus life-cycle, and is tightly regulated in the different epithelial layers. Viral gene expression may in due course trigger a successful immune response, leading to disease regression. It is thought that persistent infection, with limited expression of the viral genome, can occur following regression. It has been suggested that changes in the efficiency of immune surveillance may allow for subsequent reactivation of the disease.

Despite the differences in their epithelial tropisms and pathogenicity, all HPV types have a similar genome arrangement and gene functions [13]. Several viral proteins have been considered as targets for anti-HPV therapies. One of the major difficulties, however, is that apart from the viral E1 helicase, HPV proteins lack intrinsic enzymatic activity. The drugs currently used in the clinic to help in the treatment of HPV infections do not specifically target HPV protein function [14,15,16,17,18], and while the potential of anti-viral HPV drugs has been realised for decades, no specific anti-HPV drug has yet made it into routine clinical use [19,20]. There are also few direct viral targets, and any evaluation of treatment efficacy must take into account the possibility of spontaneous regression. HPV has developed sophisticated interactions with host cellular molecular machinery, and requires a fully differentiated epithelium to complete its productive viral life cycle (Figure 1), which introduces an added layer of complexity for in vitro research studies. Reconstituting the productive HPV life cycle in vitro is a costly and time-consuming process, and as an alternative, there are only a few appropriate animal model systems, the best of which only approximate the important PV disease associations that occur in humans. As a result, no single drug treatment has yet been shown to have a high success rate in eliminating HPV infections.

One advantage of developing treatments is that HPV infects the squamous epithelium that covers the surface of the body, which means that any potential anti-HPV drug candidate can be administered topically. Topical administration is considered an effective method of drug delivery, especially for HPV-associated lesions, because it also avoids first-pass effects in the liver, which makes it possible to administer drugs whose side effects are too severe to be administered systemically. In fact, the anticancer drugs 5-FU and Bleomycin have been used topically or by local injection in cases of verruca vulgaris [6]. For condyloma acuminatum, imiquimod, a ligand for TLR7, has been used topically and shown efficacy (Table 1). However, topical application also has limitations in relation to the size of the compounds used. Small molecules with over 500 Da molecular weight, if a physicochemical barrier of stratified epithelium is intact, are not considered to penetrate sufficiently through the skin and are not effectively delivered to the target site [21]. For example, Tacrolimus, with a molecular weight of around 800 Da, is used topically in atopic dermatitis, where the skin barrier is compromised; however, Cyclosporine, another calcineurin inhibitor with a molecular weight of around 1200 Da, is not used topically even in atopic dermatitis. Although there are many reports which describe the use of absorption enhancers to improve penetration [22], many of them have safety issues, and the design of topical formulations requires experience and know-how, as there are many ways in which drugs, base materials, and absorption enhancers can be combined.

## 3. The E6 and E2 Proteins—Primary Anti-Viral Targets of Persistent HPV Infection

HPVs primarily infect stratified epithelial tissue and cause chronic infections. To maintain a persistent infection for years or decades at stratified epithelial sites, HPV needs to infect basal cells and form a reservoir of infected cells in the epithelial basal layer. Infected cells must remain in the basal layer for prolonged periods of time alongside their uninfected neighbours, and maintain the viral genome in those cells without detection. HPVs are thought to confer such properties on the cells they infect as a result of expression of the viral E2 and E6 proteins [49] (Figure 1), and because of this, these viral proteins and their functions are the potential targets for anti-HPV therapies.

E2 is a transcriptional repressor that directly regulates E6 and E7 gene expression by binding to the viral promoter region, but E2 also modulates viral genome replication and partitioning (i.e., maintenance replication) during persistent infection [50]. In cells of the epithelial basal layer, the viral genome is replicated synchronously with the host cell genome. Although the role of E1 and E2 in viral genome amplification is well-established, HPV is thought to employ two different modes of replication; E1-dependent and E1-independent replication [49,51,52,53] during the maintenance replication phase. Although little is known about the underlying mechanisms of switching between these two replication modes, the effectiveness of E1 inhibitors as anti-viral drugs may be restricted, as they would not be expected to inhibit E1-independent HPV replication in long-lived basal cells. To develop anti-HPV therapies, it seems essential to target the preferential persistence of the infected cells in the basal layer, which all studies of viral maintenance replication suggest is dependent on E2.

The primary function of the E7 protein is to drive cells’ S-phase cell cycle re-entry in the upper epithelial layers, which is important for viral genome amplification in differentiated cells [13,54]. The E7 proteins of the high-risk α-PV types are known to target the degradation of the retinoblastoma protein family members (pRb, p107, p130) via the proteasome pathway [55], with early microarray studies identifying the downregulation of differentiation-related genes as a function of the HPV E6 proteins [56]. In tissue culture experiments, keratinocytes expressing HPV E6 but not E7 exhibit an increased proliferation rate, grow to higher cell saturation density, and exhibit a delayed commitment to differentiation [57]. E6 therefore appears to play a major role in driving basal cell persistence following infection, and as a result of increased proliferation and reduced differentiation, acts to increase cellular fitness. Indeed, cells expressing E6 are predicted to gradually displace adjacent uninfected keratinocytes over time, and to give rise to a clonal infection centre that can support the development of subsequent disease. When taken together, the viral E6 and E2 genes seem to be the most relevant anti-viral targets during persistent infection. Interestingly, it has been reported that memory helper T-cells specific for E6 and E2 (but not E7) are prominent in the general population, which might be expected if E6 and E2 are required for chronic basal cell persistence [58,59]. In the following sections, we will focus on the role of E6 during persistent infection and the therapeutic implications.

## 4. Understanding Epithelial Homeostasis in the Basal Layer

**Figure 2:**
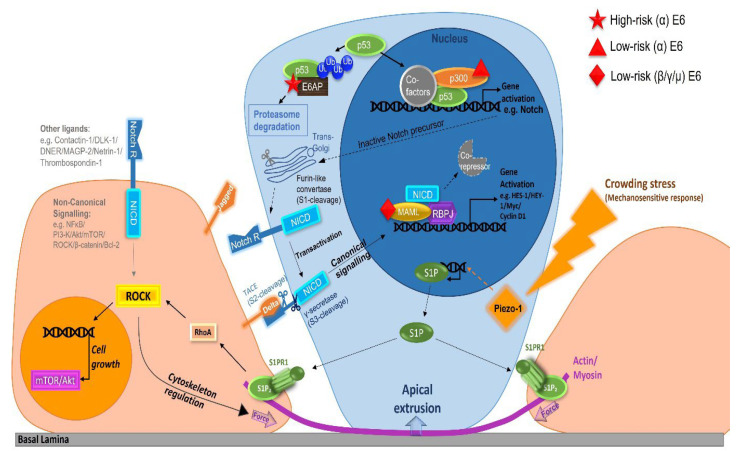
Cell Competition model of Apical Extrusion in Response to Basal Cell Overcrowding [65,66]. Upregulation and translocation of the stress-response protein Piezo-1 stimulate the release of the sphingosine-1-phosphate (S1P) molecule to adjacent cells. The binding of S1P to its receptor leads to induction of the ROCK pathway, with actin cytoskeleton contraction, cell proliferation in the surrounding cells, and cell extrusion. The cell that delaminates from the basal layer has transiently higher expression of the Notch receptor on the cell surface, and becomes a signal-receiving cell Notch pathway signal-receiving cell. p53 is one of the transcriptional regulators of the Notch precursor protein. High-risk E6 protein interacts with E6AP and targets p53 for proteasome degradation, downregulating the total Notch receptor expression level. Low-risk E6 proteins can inhibit p300 and alter p53-dependent gene transcription. Cutaneous low-risk E6 protein binds with MAML to inhibit Notch-pathway activation. ROCK can also be activated by a non-canonical Notch signalling pathway.

Epithelial development and maintenance require cellular attachment to the basal lamina, cell–cell contact inhibition as a way to regulate cell density and proliferation, and in due course, delamination of cells from the basal layer that are destined to differentiate. Understanding epithelial homeostasis and how papillomaviruses modulate it are thus of considerable importance for the development of effective treatments. Contact inhibition classically regulates proliferation in multicellular colonies, a process whereby cultured cells stop dividing when they become confluent and fully occupy a 3D space [60]. In monolayer tissue culture studies, contact inhibition of cell proliferation is marked by a plateau in the growth curve as cells reach confluency [61]. This behaviour represents proliferative arrest in most epithelial tissues, and typically leads to terminal cell differentiation and death. Contact inhibition is therefore critical in defining proper tissue architecture. Secondly, the apical extrusion of a basal epithelial cell depends on the biophysics of the epithelium and the cell “fitness”. This process has been observed in human colon epithelium, zebrafish epidermis, and Drosophila larvae epithelium [62]. As postulated by the competition theory, cells that proliferate slower are more likely to be extruded from the basal layer. In normal epithelia, faster-proliferating cells will be smaller in size than slow-cycling cells [63]. When the basal cell population is overcrowded, larger cells with greater mechanical deformation on the plasma membrane will follow delamination as a cell fate. This process is governed by a membranous mechanosensitive ion channel [64]. The cell is extruded towards the suprabasal layer by producing Sphingosine 1-Phosphate (S1P) to activate adjacent cell ROCK-dependent actin contraction (Figure 2) [65]. Once started, delamination from the basal lamina leads to activation of the Notch pathway, with keratinocytes triggering irreversible steps required for terminal differentiation. This process is particularly important for the development of anti-viral therapies. By identifying the key viral contributors that regulate the retention of the infected basal cell in the epithelium, there is the possibility that the process can be functionally modified to drive clearance of the viral reservoir from infected tissue sites.

The host machinery for epithelial homeostasis is broadly conserved among different epithelial sites. HPVs have co-evolved with their host and become adapted to their respective niches, and their E6 proteins now target similar pathways in infected cells in subtly different ways (Figure 2). The α-HPV E6 proteins, including both low- and high-risk types, target the p53 pathway to regulate transcription of the genes involved in cell cycle arrest and differentiation [67]. While the hrHPV16 E6 directly targets p53 for proteasomal degradation [68], lrHPV11 E6 also represses p53 transactivation and p53-dependent transcription by repressing p300-dependent acetylation [69]. In epithelial tissue, expression of p53 is mainly restricted to the basal layer [70], and sustained p53 induction, but not pulse induction, has been shown to upregulate the expression of genes associated with terminal cellular fates [71]. One of the direct targets downstream of p53 transactivation is the Notch receptor. Activation of the canonical Notch signalling pathway is a central regulator for basal cell commitment to differentiation, while differential interaction with ligands can define cell fate during embryonic development [72]. The hrHPV16 E6 protein not only downregulates total Notch receptor expression levels in a p53-dependent manner [57], but also changes the surface ligand distribution to favour DLL4 expression [73]. These molecular modifications make sense when considered with the phenotypes of increased proliferation and a delayed commitment to differentiation mediated by hrHPV16 E6 expression.

By contrast, the way that other cutaneous lrHPV E6 proteins (β, γ, µ and η genera) affect basal cell commitment to differentiation is not as direct as seen with the α-HPV types, which modulate total Notch receptor levels via p53. LrHPV types from outside of the α-group modify the host cellular environment through the Notch-MAML-dependent pathway (Figure 2). The E6/MAML-association is important for μ-PV, β-HPV and γ-HPV pathogenesis [74,75,76]. MAML mutant cells can gradually displace their wild-type basal cell neighbours in mice as a result of their increased proliferation rate and reduced ability to commit to differentiation [77]. These observations support our hypothesis of an evolutionarily conserved role for the HPV E6 proteins in regulating the host cellular environment for long-term persistence within the basal epithelium. The dependence of papillomaviruses on ancient pathways, which are conserved across different species and epithelial sites, suggests that a common antiviral strategy may facilitate the clearance of infected cells from the body.

## 5. Experimental Models to Evaluate and Modulate E6 Function during HPV Persistence

**Figure 3:**
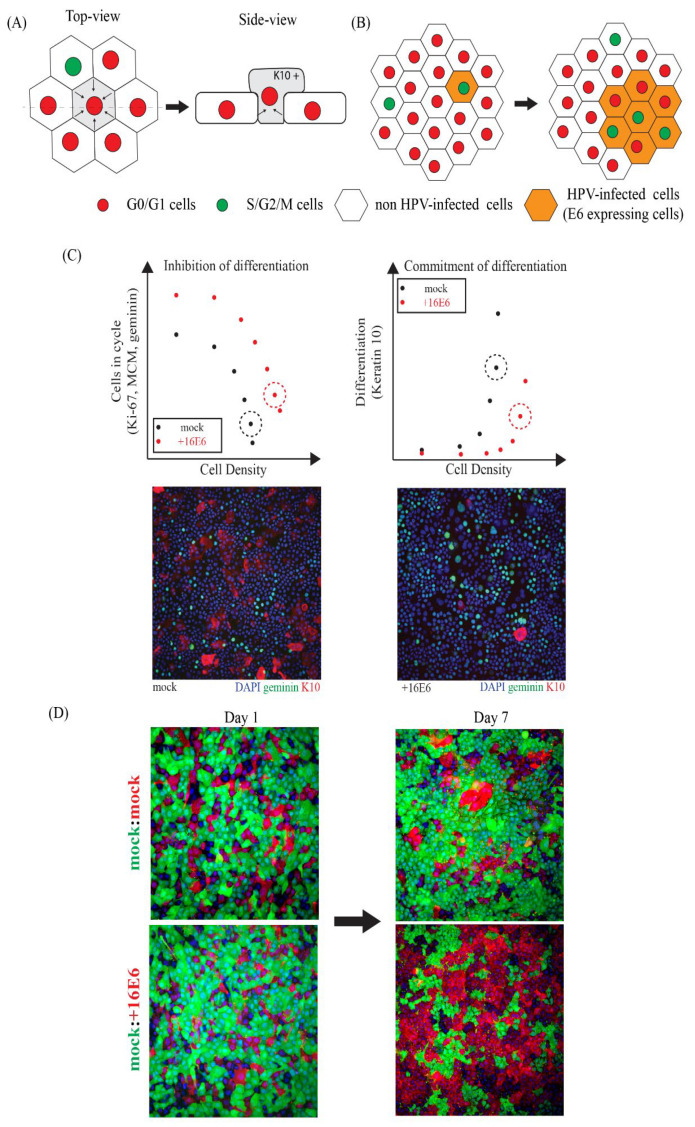
Basal Layer Epithelial Homeostasis in the Presence or Absence of hrHPV E6 Protein. (**A**) In the uninfected basal layer, keratinocytes exit the cell cycle (at confluence) in response to contact inhibition signals, with a small proportion of cells retaining their proliferative potential. Keratinocytes that stochastically commit to differentiate and delaminate from the basal layer and are extruded into the upper layers where they express keratinocyte differentiation, such as keratin 10 (K10). (**B**) The expression of viral E6 protein confers a preferential advantage over neighbouring uninfected cells with regard to basal layer retention. HPV-infected keratinocytes (expressing E6, orange) will gradually displace uninfected cells (white) as a result of their altered response to the local cellular environment. (**C**) As cell density increases, keratinocytes exit the cell cycle and express the differentiation marker (e.g., K10). The proportion of cells in cycle can be measured using cell cycle markers (geminin, Ki67, or MCM). As cell density increases, E6-expressing cells remain in cycle and differentiation (K10 expression) is inhibited. The dashed circle represents the point at which the cultured keratinocytes reach full confluency. (**D**) The images show two fluorescently tagged cell lines immediately after plating, and again seven days later. The upper images reveal the equivalent growth of red and green tagged normal keratinocytes (eGFP)/(mCherry). The lower images reveal how the preferential growth advantage of cells expressing E6 is manifest over the seven-day growth period (eGFP)/HPV16E6(mCherry). Cells expressing HPV16E6 are preferentially retained in the lower layer of cells and displace normal keratinocytes.

To date, anti-HPV therapies that target E6 have been evaluated with respect to their cytotoxicity and their role in stimulating apoptosis in HPV-associated cancer cell lines [78]. Although E6 is often considered as a viral oncogene which contributes to HPV carcinogenesis, the primary role of the E6 protein during productive infection is to modulate normal epithelial homeostasis to facilitate persistent infection. E6 confers an enhanced proliferative potential on infected basal cells, as well as a delay in commitment to differentiation, and preferential basal layer persistence when compared to uninfected cells. In in vitro experiments, uninfected keratinocytes leave the cell cycle and commit to differentiation as their cell density and their contacts with surrounding cells increases (Figure 3A). We can observe and quantify the degree of contact inhibition and early differentiation by counting the nuclear density, and by staining for cell cycle markers, such as MCM, Ki67 and geminin, along with the early differentiation marker keratin 10 (Figure 3B). In the presence of E6 expression, keratinocytes are maintained in the cell cycle, and show a delay in the expression of differentiation markers even at high cell density (Figure 3B). Preferential persistence in the basal layer, of E6 expressing cells, has been investigated here using a cell competition assay. Cells that express mCherry or eGFP are seeded at high (confluent) density on day 0. If cells have no advantage over each other, then the ratio of mCherry to eGFP-expressing cells will be maintained over time. By contrast, mCherry cells expressing E6 have an advantage over their “normal” neighbours, allowing the ratio to gradually increase over time (Figure 3C). Such approaches allow candidate anti-HPV molecules to be assessed for their ability to suppress the phenotype of E6-expressing cells and to bring them more in line with those of normal keratinocytes. Such innovative approaches have the potential to identify drugs or drug combinations which may go further than this, and confer a growth disadvantage on cells expressing HPV genes, which is expected to lead to the development of clinically useful anti-viral HPV therapies in due course.

## 6. Conclusions

Undoubtedly, the availability of prophylactic vaccines and the implementation of screening programmes have successfully reduced the burden of hrHPV infections and associated diseases, including cervical cancer. However, these efforts do little for those who are infected by non-vaccine HPV types or who have not been vaccinated, or for individuals infected by the prevalent lrHPV types, which continue to impose a considerable economical and medical burden on the public. The development of anti-HPV therapies remains a pressing issue if HPV-associated morbidity and mortality are to be eliminated.

A primary role of the E6 protein during productive infection is to interfere with normal epithelial homeostasis to facilitate viral persistence. In normal stratified epithelium, basal cells respond to contact inhibition of proliferation to maintain an appropriate and balanced basal cell density. Cells that are more sensitive to mechanical deformation by crowding stress will be extruded towards the apical epithelium, signalling the cytoskeleton of the adjacent cell to contract, and for ongoing neighbouring cell proliferation to force its loss from the epithelial basal layer. The delaminating cell must commit to differentiation by activating the Notch signalling pathway in order to maintain the stratified epithelium. To retain infected keratinocytes at the basal layer, HPV encodes its E6 protein to alter the process of contact inhibition of proliferation, cellular junction formation, and commitment to differentiation. For clearance of HPV infection, we suspect that the mechanisms that allow persistence of viral reservoir must be understood at a molecular level, and in due course, targeted by anti-virals. Specific in vitro models of the basal and spinous layer of the epithelium open the way for future investigation of how HPV regulates normal epithelial homeostasis, and will facilitate the development of “true” anti-HPV therapies.

## Figures and Tables

**Table 1:** Summary of current HPV treatments and (**A**) HPV protein function and anti-viral agents identified from research studies; (**B**) agents currently in clinical use.

**(A)**		
**Viral Proteins**	**Function in the Viral Life Cycle**	**Proposed Drugs and Mechanism**
E1	DNA binding protein, helicase, ATPase, viral genome amplification	Low-risk E1 helicase inhibitors [23]
		Inhibitors of tyrosyl-DNA phosphodiesterase 1 and PARP1 [24]
E2	Transcriptional regulation of early genes, initiation of DNA replication and partitioning the viral genome	Indandione inhibitors reversibly bind to the transactivation domain of E2, inhibition of E1/E2 interaction [25,26]
		Pyrrole–imidazole polyamides bind to viral DNA and prevent E1-E2 initiation of replication, with potential modification by computational methods [27,28,29,30]
E4	Helping genome amplification, virus synthesis, might have an additional role during virus release and transmission	Not considered as a drug target
E5	Only encoded by α-HPVs, transforming activity in vitro, role in viral immunosurveillance	Not considered as a drug target
E6/E7	Oncogenes, modify cell cycle status for viral genome amplification in the differentiated epithelium, role in viral persistence	Small interfering RNA (siRNA) downregulate E6/E7 mRNA levels and induces cancer apoptosis [31,32,33,34,35]
		Antisense deoxynucleotides [36]
		Ribozymes [37]
		Intrabodies target E6 protein [38,39]
		Flavonoid-derived compounds disrupt E6–E6AP binding [40]
		Other small molecules inhibit E6 and cellular protein interaction [41,42]
		Proteasome inhibitors (or combination therapies) prevent p53 degradation by E6 [43,44]
		HIV protease inhibitors prevent E6-mediated p53 degradation in HPV-positive cells [45,46,47]
		Histone deacetylase (HDAC) inhibitors revert E7 oncogenic functions [48]
(**B**)		
**Clinically used drugs**	**Class**	**Mode of action**
Retinoid (systemic)	Vitamers of vitamin A	Disrupts epidermal growth and differentiation [15]
Salicylic acid (topical)	Organic compound	Salicylic acid is keratolytic, destroying the virus-infected epidermis. It may also stimulate an immune response [16]
5-FU, Bleomycin	Anticancer drug	Antimitotic; thymidylate synthase inhibitor (5-FU) & induction of DNA strand breaks (Bleomycin) [14,18]
Imiquimod	Innate immune system stimulator	Toll-like receptor 7 agonist [17]

## Data Availability

Not applicable.
